# Early-Diagnosed Gestational Diabetes Mellitus: Molecular and Metabolic Evidence Before 24 Weeks of Gestation

**DOI:** 10.3390/biomedicines14102340

**Published:** 2026-10-14

**Authors:** Gulshat Bilyalova, Aida Baibusunova, Assel Boshanova, Zhansulu Akylzhanova, Alisher Idrissov, Aigul Turgumbayeva, Damir Biktashev, Assel Baubekova, Saltanat Kulbayeva, Dinara Azanbayeva, Dinara Turzhanova, Cholpon Stakeeva, Ruslan Alexeyev, Anar Kystaubayeva

**Affiliations:** 1 Department of Obstetrics and Gynaecology No. 2, Astana Medical University, Astana 010000, Kazakhstan; 2 Department of Internal Diseases No. 2, Astana Medical University, Astana 010000, Kazakhstan; 3 Department of Obstetrics and Gynaecology No. 1, Astana Medical University, Astana 010000, Kazakhstan; 4 Department of Obstetrics and Gynecology, South Kazakhstan Medical Academy, Shymkent 160019, Kazakhstan; 5 Department of Dermatology, Astana Medical University, Astana 010000, Kazakhstan; 6 Department of Sports Medicine, Rehabilitation and Clinical Research, Kazakh National University of Sports, Astana 010000, Kazakhstan; 7 I.K. Akhunbaev Kyrgyz State Medical Academy, Bishkek 720020, Kyrgyzstan; 8 Viamedis LLP, Astana 010000, Kazakhstan; 9 Multidisciplinary City Hospital No. 3, Astana 010000, Kazakhstan

**Keywords:** early gestational diabetes, insulin resistance, beta-cell compensation, adipokines, gut microbiota, placenta, trophoblast, extracellular vesicles

## Abstract

Gestational diabetes mellitus (GDM) is conventionally diagnosed at 24–28 weeks of gestation, although hyperglycaemia may be detected earlier. Early gestational diabetes mellitus (eGDM), defined here as hyperglycaemia diagnosed before 24 weeks in the absence of overt diabetes, is characterised by limited but emerging molecular and metabolic evidence. In women with eGDM diagnosed before 20 weeks, DALI phenotyping identified insulin-resistant, secretion-dominant and mixed phenotypes using HOMA-IR and the OGTT-derived Stumvoll first-phase index, with greater obstetric risk in the resistance-dominant group. In the TOBOGM cohort, eGDM was associated with higher diacylglycerol and fatty-acid species, which remained associated with eGDM and glycaemic indices after adjustment for clinical risk factors. Early-pregnancy SHBG provides additional evidence, with a further reduction reported in women diagnosed before 20 weeks. The principal limitation is that eGDM-specific evidence remains sparse. Most early-pregnancy biomarker studies measured women before 24 weeks but diagnosed GDM later and therefore describe antecedents of subsequent disease rather than eGDM diagnosed before 24 weeks. Most mechanistic evidence also derives from conventional-window GDM or placental tissue obtained at delivery, preventing determination of whether reported abnormalities precede, accompany or result from early hyperglycaemia. Longitudinal studies with early diagnosis, concurrent assessment of insulin sensitivity and secretion, adjustment for adiposity and independent validation are therefore needed to define the molecular phenotype of eGDM.

## 1. Introduction

Hyperglycaemia in pregnancy affected an estimated 21.1 million live births in 2021, mostly because of GDM rather than pre-existing diabetes [1]. The consequences outlast delivery: subsequent diabetes is markedly more frequent after GDM [2], cardiovascular events are increased even among young women [3], and in the HAPO Follow-up Study maternal GDM was associated with higher childhood adiposity and altered childhood glucose metabolism [4,5].

The conventional 24–28-week diagnostic approach was informed substantially by HAPO, in which oral glucose tolerance tests were performed at 24–32 weeks of gestation, with 28 weeks as the target. HAPO related fasting, 1 h and 2 h glucose values below overt-diabetes thresholds to adverse perinatal outcomes in more than 23,000 pregnancies [6], and those associations shaped the IADPSG and WHO criteria for hyperglycaemia first detected in pregnancy [7,8].

The Treatment of Booking Gestational Diabetes Mellitus (TOBOGM) trial randomised 802 women with GDM diagnosed before 20 weeks, at a mean gestational age of 15.6 ± 2.5 weeks, to immediate or deferred treatment, and immediate treatment reduced the composite adverse neonatal outcome from 30.5% to 24.9% [9]. Two 2025 meta-analyses of randomised trials found no consistent benefit across most maternal and neonatal endpoints, with reduced neonatal respiratory distress and, in one sensitivity analysis, less macrosomia [10,11]. In a treated cohort, the 68 women whose GDM was diagnosed before 12 weeks had rates of macrosomia, large-for-gestational-age birth and neonatal intensive-care admission (21.8%, 39.6% and 38.5%) that did not differ significantly from those of 65 women with pre-existing diabetes; with fewer than 70 women per group, this indicates an absence of demonstrated difference rather than equivalence [12]. Early detection therefore identifies a high-risk group whose residual risk is not abolished by glucose-lowering treatment, which is an argument for examining what is abnormal in these pregnancies.

Existing reviews of eGDM address diagnostic thresholds and the value of early treatment [13], the individualisation of therapy [14] and the global burden of early hyperglycaemia [15]. To our knowledge, the molecular evidence has not been organised according to the gestational age at which samples were obtained and at which GDM was diagnosed, or according to whether proposed early biomarkers remain associated with GDM after adjustment for maternal body-mass index (BMI). Both considerations matter, because much of the relevant molecular literature is based on term placental tissue or on women diagnosed at 24–28 weeks, and maternal adiposity is a potential confounder for many candidate biomarkers.

This narrative review synthesises molecular and metabolic evidence relevant to eGDM diagnosed before 24 weeks. Each finding is assigned to one of four evidence classes according to gestational age at sampling and at diagnosis and the material studied (Section 3), so that eGDM diagnosed before 24 weeks, early predictors of later GDM, conventional-window GDM and mechanistic evidence are kept separate. Within this framework, the review considers maternal metabolic, dietary, gut-microbial, placental, trophoblast and extracellular-vesicle findings, their current clinical implications, and the study designs needed to determine whether reported changes precede or accompany hyperglycaemia.

## 2. Review Design and Literature Search

This is a narrative review supported by a literature search. The search aimed to identify the principal molecular and metabolic evidence relevant to the pre-24-week window and to classify it by gestational age at sampling and at diagnosis.

PubMed/MEDLINE, Embase and Web of Science were searched from inception to 31 July 2026, without language restriction, and reference lists of included articles and recent reviews were screened by hand. Terms combined population and window (gestational diabetes, early or early-onset GDM, eGDM, hyperglycaemia in pregnancy, first trimester, booking, before 20 weeks, before 24 weeks) with measurement domain (insulin resistance, HOMA-IR, QUICKI, disposition index, beta-cell function, adipokines, sex hormone-binding globulin, triglyceride-glucose index, proteomic, metabolomic, lipidomic, extracellular vesicle, gut microbiota, trophoblast, placental transporter, mTOR).

Eligible reports were original human studies of any design that measured a metabolic, endocrine, inflammatory, microbial, omic, placental or extracellular-vesicle variable in relation to GDM or eGDM and reported, or allowed derivation of, gestational age at sampling and at diagnosis. Systematic reviews and meta-analyses were used for context, and placental, cellular and animal studies were included only as mechanistic evidence. Studies of pre-existing diabetes, abstracts without full data and reports in which the timing of sampling or diagnosis could not be established were excluded; when several reports came from one cohort, the report most directly addressing the early window was cited.

Titles and abstracts, and then full texts, were screened independently by two authors, with disagreements resolved by discussion with a third author. Data on design, sample size, gestational age at sampling and at diagnosis, diagnostic criteria, handling of adiposity, effect estimates and validation were extracted by one author and checked by a second.

Because designs, diagnostic criteria, analytical platforms and outcomes were heterogeneous, no formal risk-of-bias score was assigned. Each study was instead appraised on four features that determine what it can show about eGDM: its evidence class, whether sampling preceded or coincided with diagnosis, how adiposity was handled (adjustment, matching, stratification or none) and whether discrimination was validated in an independent sample.

## 3. Defining Early Gestational Diabetes Mellitus and Classifying the Evidence

No universally accepted, outcome-derived definition of early GDM exists; the term has been applied to hyperglycaemia diagnosed in the first trimester, before 20 weeks or before 24 weeks, using different glucose thresholds. In this review, eGDM is an operational term rather than an established diagnostic category: hyperglycaemia diagnosed before 24 weeks that does not reach thresholds for overt diabetes in pregnancy (fasting plasma glucose ≥ 7.0 mmol/L, 2 h plasma glucose ≥ 11.1 mmol/L after a 75 g oral glucose tolerance test [OGTT], random plasma glucose ≥ 11.1 mmol/L with symptoms, or HbA1c ≥ 6.5% where HbA1c is incorporated) [7,8].

American Diabetes Association guidance separates three early-pregnancy categories that are often conflated. Non-pregnancy thresholds applied before 15 weeks identify previously unrecognised diabetes, classified as diabetes complicating pregnancy rather than GDM; early abnormal glucose metabolism is defined as fasting plasma glucose 6.1–6.9 mmol/L (110–125 mg/dL) or HbA1c 5.9–6.4% before 15 weeks and is associated with adverse outcomes and later GDM; and screening at 24–28 weeks remains the conventional window for other women [16]. Studies of early hyperglycaemia variously apply IADPSG/WHO 75 g thresholds before 24 weeks, fasting cut-offs of ≥5.1 or ≥5.6 mmol/L, or Carpenter–Coustan values within a two-step strategy. Because fasting glucose falls physiologically in early pregnancy, the prognostic meaning of thresholds derived for late pregnancy cannot be assumed in early gestation.

Human placental lactogen is detectable from about 6 weeks, and its insulin-antagonist effect becomes prominent only from the mid-second trimester [17]. In a TOBOGM-linked cohort including women diagnosed both before 20 weeks and in the conventional window, early-pregnancy human placental lactogen did not differ between women who did and did not develop GDM, and lower rather than higher hPL was independently associated with higher fasting glucose, which argues against exaggerated placental hormone output as an explanation for hyperglycaemia at this stage [17].

The diagnosis is also unstable. Between 30% and 50% of women who screen positive before 20 weeks have normal glucose tolerance when retested at 24–28 weeks [13]. This range plausibly reflects diagnostic threshold, gestational age at testing and population characteristics together, but their contributions have not been quantified separately because few cohorts report retesting. Threshold and glycaemic severity are best documented: in the deferred-treatment arm of TOBOGM, 121 of 367 women (33%) diagnosed before 20 weeks by WHO 2013 criteria no longer met criteria at 24–28 weeks; regression was more likely in the lower than in the upper glycaemic band (adjusted odds ratio 3.09, 95% CI 1.87–5.10), affected about half of lower-band cases, and was best predicted by the early 1 h glucose value [18]. Population characteristics also contribute, since polycystic ovary syndrome was associated with persistence [18], whereas the effect of gestational age at testing, although plausible given the early physiological fall in fasting glucose, has not been examined directly. Women whose early GDM regressed had outcomes similar to those without GDM [18]. Cohorts assembled before 20 weeks therefore contain persistent and transient hyperglycaemia in unknown proportions unless retesting is reported, and diagnosis before 12 weeks identifies more severe glycaemia and more diabetes-related complications than later cut-offs [12,19].

To keep these distinctions explicit, evidence is assigned to four classes, indicated in the text and in Table 1 and Table 2. Class A (early-diagnosed GDM): hyperglycaemia diagnosed before 24 weeks, with the measurement made in the same early window; because a substantial proportion of women diagnosed early are normoglycaemic on retesting at 24–28 weeks, this class denotes the timing of diagnosis and does not imply persistent hyperglycaemia. Class B (antecedents of later GDM): measurement before 24 weeks but GDM diagnosed at 24–28 weeks; such studies identify predictors of subsequent GDM, not features of early-diagnosed GDM. Class C (conventional-window GDM): measurement and diagnosis at or after 24 weeks. Class D (mechanistic evidence): placental or adipose tissue obtained at delivery, cell systems and animal models, which cannot place an abnormality before or after the onset of maternal hyperglycaemia. Cohorts combining early and later diagnoses are labelled as mixed.

**Table 1:** Metabolic and physiological features of normal early pregnancy as well as early-window and conventional-window GDM, with evidence class, gestational age at sampling and at diagnosis, and handling of adiposity.

Feature	Normal Early Pregnancy	Early-Window Finding (<24 wk)	Evidence Class; GA at Sampling/at Diagnosis; Adiposity	Conventional-Window GDM (≥24 wk)
Insulin sensitivity, fasting surrogate	Preserved or slightly increased at 12–14 wk relative to pre-pregnancy [20,21,22]	HOMA-IR raised and QUICKI reduced; HOMA-beta not different [23]	Class B; 6–12 wk/24–28 wk; stratified by first-trimester BMI and adjusted	Markedly reduced by mid-to-late gestation [21,22]
Insulin sensitivity and secretion, OGTT-derived	At ≤15 wk, Stumvoll-estimated secretion augmented independently of Matsuda sensitivity, which was slightly lower than postpartum [24]	Resistance-dominant, secretion-dominant and mixed phenotypes by median split on HOMA-IR and the Stumvoll first-phase index; resistance-dominant carried the greater obstetric risk [25]; in a second cohort, early GDM showed markedly reduced sensitivity and no sensitivity-adjusted augmentation of secretion [24]	Class A; <20 wk/<20 wk (DALI; (restricted to BMI ≥ 29 kg/m^2^) and ≤15 wk/≤15 wk (SPRING; BMI-adjusted)	Secretion fails to match rising insulin resistance [26,27]
Beta-cell response	First-phase insulin response increased at 12–14 wk (intravenous glucose tolerance test) [22]	Fasting HOMA-beta not raised despite higher HOMA-IR [23]; Stumvoll index not referenced to concurrent sensitivity in DALI [25] but referenced to Matsuda sensitivity in SPRING [24]	Class B (HOMA): 6–12 wk/24–28 wk; class A (Stumvoll): <20 wk/<20 wk	Inadequate compensation for insulin resistance [26,27,28]
Placental anti-insulin hormone exposure	hPL detectable from ~6 wk and rising, below third-trimester concentrations [17]	Early hPL not different between women who did and did not develop GDM [17]	Mixed (A and B); <20 wk/early or 24–28 wk (TOBOGM-nested cohort); adiposity handling not available	Approaching maximal insulin-antagonist effect [17]
Adipokines	Reference state	Lower adiponectin and sOB-R, higher FABP4 [29,30,31]	Class B; 1st trimester or 10–14 wk/24–28 wk; adjusted for BMI [30,31]	Low adiponectin; inflammatory markers raised in tissue at delivery (class D) [32,33]
Hepatic and androgen markers	Reference state	SHBG 3.7% lower, free androgen index 4.6% higher; SHBG a further 6.7% lower in women diagnosed <20 wk [34]	Mixed (subgroup class A); ~10.7 wk/any gestation, subgroup < 20 wk; adjusted for pre-pregnancy BMI	Not the window in which these estimates were generated
Lipid handling, glucose-lipid index	Physiological rise in triglycerides	Higher TyG index; adds little discrimination beyond age and BMI [35,36]	Class B; 1st trimester (<14 wk)/24–28 wk; adjusted for pre-pregnancy BMI [36]	Established dyslipidaemia at diagnosis [37]
Lipid handling, lipidomic	Not characterised before 20 wk	Higher diacylglycerols and fatty acids, lower lysophosphatidylcholines [38]	Class A; <20 wk/<20 wk (TOBOGM); adjusted for BMI and clinical risk factors; not replicated	Established dyslipidaemia at diagnosis [37]
Gut-derived metabolites	Not systematically characterised before 20 wk	Choline associated with higher, betaine and carnitine with lower GDM risk per SD; TMAO not associated [39]	Class B; 12–16 wk/24–28 wk; adjusted for early-pregnancy BMI	Microbial and inflammatory differences, detectable prospectively months before diagnosis [40,41]
Placental and trophoblast data	First-trimester GLUT4 expression in syncytiotrophoblast [42]	Limited to placenta-derived exosome proteomics [43]	Class B; 12–16 wk/24–28 wk; adiposity handling not available	Transporter, mTOR, mitochondrial and ER findings, almost all in delivered tissue (class D) [44,45,46,47,48]
Glucose levels	Reference state	About one-third (TOBOGM) to one-half of women revert to normoglycaemia at 24–28 wk, depending on the early threshold [13,18]	Class A; screened <20 wk/retested 24–28 wk	Persists by definition at 24–28-wk testing

Evidence classes: A, early-diagnosed GDM (measurement and diagnosis before 24 wk); B, measured before 24 wk, GDM diagnosed at 24–28 wk; D, delivered tissue or experimental systems; mixed, cohorts combining early and conventional-window diagnoses. Abbreviations: ER, endoplasmic reticulum; FABP4, fatty-acid-binding protein 4; GA, gestational age; hPL, human placental lactogen; OGTT, oral glucose tolerance test; SD, standard deviation; SHBG, sex hormone-binding globulin; sOB-R, soluble leptin receptor; TMAO, trimethylamine-N-oxide; TyG, triglyceride-glucose index.

**Table 2:** Adjusted associations of proposed early-pregnancy biomarkers with GDM and eGDM: evidence class, gestational age at sampling and at diagnosis, effect estimates, covariates and adiposity handling, and predictive performance reported separately.

Biomarker	Study; Evidence Class	GA at Sampling/at Diagnosis	Adjusted Effect Estimate (95% CI)	Covariates; Adiposity Handling	Predictive Performance (Validation)
HOMA-IR	Duo et al. [23]; class B	6–12 wk/24–28 wk	Adjusted OR per unit: 2.94 (2.15–4.02) normal weight; 3.19 (2.01–5.06) overweight; 9.42 (1.71–51.77) obesity	Age, first-trimester BMI, family history of diabetes, prior adverse pregnancy outcome, gestational weight gain and serum lipid profile; stratified by first-trimester BMI	AUC 0.733 (0.701–0.765); development sample only
SHBG, testosterone, FAI	Mustaniemi et al. [34]; mixed, subgroup class A	Mean 10.7 wk/any gestation; subgroup < 20 wk	SHBG 3.7% lower (0.1–7.3); testosterone 3.1% higher (0.1–6.2); FAI 4.6% higher (1.9–7.3); SHBG a further 6.7% lower (0.7–12.7) in the <20-wk subgroup	Gestational week at sampling, pre-pregnancy BMI, maternal age	Not reported as a prediction model
Adiponectin	Williams et al. [29]; class B	Early pregnancy/later in pregnancy	OR 4.6 (1.8–11.6) for <6.4 µg/mL versus higher	Adjusted for confounders; individual covariates, including whether BMI was included, not available	Not reported as a prediction model; 41 cases, 70 controls
Adiponectin	Lacroix et al. [30]; class B	First trimester/24–28 wk	OR 1.12 per 1 µg/mL decrease; CI not available (*p* = 0.02)	First-trimester BMI and HbA1c	Not reported as a prediction model
FABP4, adiponectin, sOB-R	Francis et al. [31]; class B	10–14 wk/24–28 wk	OR highest versus lowest quartile: FABP4 4.87 (1.84–12.9); adiponectin 0.13 (0.05–0.35); sOB-R 0.24 (0.11–0.52)	Conditional logistic regression, sets matched on age, race/ethnicity and gestational week; adjusted for age, gestational week, parity and family history of diabetes; most associations persisted, slightly attenuated, after further adjustment for pre-pregnancy BMI	AUC 0.71 (0.66–0.77) to 0.77 (0.72–0.82) on adding the panel to age, race/ethnicity, family history and random glucose (BMI not included); development sample only
TyG index	Guo et al. [36]; class B	<14 wk/24–28 wk	OR 2.10 (1.72–2.56) per unit	Ethnicity, pre-pregnancy BMI, age, assisted reproduction, miscarriage history, gravidity, parity, gestational age at delivery, gestational weight gain	AUC 0.641 for TyG alone and 0.672 combined with age and BMI; development sample only
Vitronectin	Ravnsborg et al. [49]; class B	8–14 wk/24–28 wk	Adjusted estimate not reported separately from the prediction model	Maternal age and family history of diabetes (previous GDM not retained; previous macrosomia excluded); cases and controls matched on BMI; cohort restricted to BMI ≥ 27 kg/m^2^	AUC 0.798 to 0.806 on adding vitronectin; discovery *n* = 60, second sample set *n* = 210 from the same population; no external validation
Choline, betaine, carnitine	Wu et al. [39]; class B	12–16 wk/24–28 wk	Per SD: choline 1.16 (1.01–1.34); betaine 0.81 (0.70–0.95); carnitine 0.80 (0.69–0.94)	Conditional logistic regression on age-matched sets (1:4); adjusted for age, race, education, early-pregnancy BMI, physical activity, smoking, alcohol, passive smoking, sleep score, parity, disease history and the other metabolites	Not reported as a prediction model
Urinary metabolite panel	Koos and Gornbein [50]; Murphy et al. [51]; class B (6–19 wk) and class C (≥24 wk)	6–19 wk and ≥24 wk/24–28 wk	Matched design; no per-metabolite adjusted estimates reported	Matched on maternal age, pre-pregnancy BMI and GA at collection; residual confounding by adiposity not excluded	7-metabolite model, 96.7% accuracy (6–19 wk); 4-metabolite tree, 89.1% accuracy and ROC area 0.923 (≥24 wk); development samples only
Plasma lipid species (TOBOGM)	Saadati et al. [38]; class A	<20 wk/<20 wk	Diacylglycerol and fatty-acid species remained associated with eGDM and glycaemic indices; per-species estimates not reported	Maternal age, BMI, gravidity, gestational week at sampling, ethnicity, family history of diabetes	Not reported as a prediction model; not replicated
Discovery proteomic panels	Zhao et al. [52]; Liu et al. [53]; Mavreli et al. [54]; class B	11–18 wk/24–28 wk	No adjusted estimates reported	None reported; no BMI adjustment	AUC 0.94–1.00 in small discovery and verification sets; no external validation

## 4. Monogenic Hyperglycaemia Detected in the Early Window

Some women labelled as having eGDM carry a lifelong, non-progressive form of monogenic diabetes. Heterozygous inactivating variants in glucokinase (GCK-MODY) raise the glucose set-point for insulin secretion, producing a stable, mild fasting hyperglycaemia of approximately 5.4–8.3 mmol/L. Because pregnancy is often the first time glucose is measured, this condition surfaces preferentially in early, fasting-glucose-based screening. In a multicentre Portuguese cohort of 18,421 women with GDM diagnosed at any gestational age, 3.6% met the clinical criteria for suspected GCK-MODY, an estimated prevalence of 1.5% among women labelled as having GDM, and suspected GCK-MODY was associated with neonatal birthweight below the 25th percentile (odds ratio 1.23, 95% CI 1.04–1.46), which the authors attributed to excessive insulin treatment of mothers whose foetuses may carry the same variant [55]. GCK-MODY does not remit after delivery and does not share the acquired metabolic features discussed below, so its inclusion dilutes eGDM cohorts; fasting hyperglycaemia at a normal pre-pregnancy BMI, particularly with a parental history of mild diabetes, is a reason to consider monogenic testing.

## 5. Metabolic Adaptation in Normal Early Pregnancy

Early gestation is not uniformly insulin resistant. Longitudinal studies show that women with GDM follow a different trajectory of insulin action from women with normal glucose tolerance and that the divergence is not confined to late gestation [20,21]. In 34 women studied by intravenous glucose tolerance testing and euglycaemic clamp before pregnancy, at 12–14 weeks and at 34–36 weeks (19 with normal glucose tolerance and 15 who developed GDM), first-phase insulin response rose from 465.1 ± 268.5 before pregnancy to 720.0 ± 358.2 in early pregnancy, while insulin sensitivity rose from 0.10 ± 0.04 to 0.12 ± 0.05 and then fell to 0.06 ± 0.03 in late pregnancy, and the early augmentation of secretion persisted after adjustment for insulin sensitivity and GDM status [22]. A contemporary cohort using OGTT-derived indices at ≤15 weeks confirmed an augmentation of insulin secretion independent of insulin sensitivity in women without GDM [24].

At 12–14 weeks, pregnancy therefore combines preserved or slightly improved insulin sensitivity with an increased secretory response. Because the period from 15 to 23 weeks was not sampled, insulin resistance detected at 12–14 weeks is a departure from the measured normal pattern at that gestational age rather than an early instance of the late-gestation pattern, and these observations should not be generalised to the whole period before 24 weeks (Table 1).

## 6. Insulin Resistance and Beta-Cell Compensation

Two questions need to be delineated: whether insulin resistance in early pregnancy predicts GDM diagnosed later, and whether insulin resistance, inadequate secretion, or both characterise women already diagnosed before 24 weeks. Because hyperglycaemia arises only when secretion fails to match the prevailing resistance, indices that relate secretion to sensitivity are more informative than insulin concentration alone, and a woman with severe insulin resistance may have high insulin levels and still be under-compensating [26,27].

Fasting surrogates identify an abnormality well before conventional screening (class B). Among 1343 women studied at 6–12 weeks, those who later developed GDM had higher HOMA-IR and lower QUICKI, whereas HOMA-beta did not differ significantly. Within the strata of first-trimester BMI and after adjustment for age, family history of diabetes and prior adverse pregnancy outcome, adjusted odds ratios for HOMA-IR were 2.94 in normal-weight women, 3.19 in women with overweight and 9.42 in women with obesity, with an overall area under the curve of 0.733 [23]. Because GDM was diagnosed in the conventional window, these estimates describe an antecedent of later GDM, not a feature of eGDM diagnosed before 24 weeks.

HOMA-IR and HOMA-beta are not equivalent to dynamic measurements of insulin sensitivity or beta-cell compensation. Both are derived from a single fasting glucose-insulin pair using a model calibrated in non-pregnant adults; they reflect mainly basal, liver-weighted insulin action and secretion, have limited precision in individuals, depend on the insulin assay and do not capture the response to a glucose load [56]. HOMA-beta cannot be interpreted without reference to concurrent sensitivity, because a normal or raised value may still be inadequate for the prevailing resistance. The euglycaemic clamp and intravenous glucose tolerance test measure sensitivity and acute secretion directly and allow a disposition index to be calculated; they have been applied at 12–14 weeks in women with and without later GDM [22], but this search did not identify their use in women diagnosed before 20 weeks.

In early-diagnosed GDM (class A), the DALI analysis of women with a BMI of 29 kg/m^2^ or more and GDM diagnosed before 20 weeks estimated insulin resistance by HOMA-IR and secretion by the Stumvoll first-phase index, which is calculated from fasting and 30 min OGTT glucose and insulin and therefore reflects post-load secretion, although it is an empirical surrogate validated against the intravenous glucose tolerance test rather than a direct measurement [25]. Women classified by median split as resistance-dominant carried greater obstetric risk than those with isolated low secretion [25]. Three limitations apply: the cohort was confined to women with obesity; categories defined by median split depend on the cohort’s own distribution, so the same woman could be classified differently elsewhere; and secretion was not referenced to concurrent sensitivity as a disposition index. These subgroups are therefore provisional rather than stable treatment strata. The SPRING cohort partly addresses the missing comparison: OGTTs were performed at ≤ 15 weeks, at 24–32 weeks and postpartum in 166 women with diabetes risk factors, 21 of whom met IADPSG criteria for GDM in early pregnancy (class A). In BMI-adjusted models, women with early GDM had markedly lower insulin sensitivity (Matsuda index) in early pregnancy than postpartum (β = −0.59) and, unlike women without GDM, showed no early-pregnancy augmentation of insulin secretion adjusted for sensitivity or of the pregnancy insulin physiology index, a disposition-type measure [24]. Because this evidence comes from a small early-GDM group in one cohort and relies on OGTT-derived surrogates, whether secretion is inadequate for the prevailing sensitivity in eGDM still requires confirmation. The hepatic, adipose and skeletal-muscle components of the resistance-dominant pattern have not been measured separately in eGDM.

In the conventional window (class C), GDM is framed as failure of beta-cell compensation for pregnancy-induced insulin resistance [26], and declining compensation after GDM tracks progression toward type 2 diabetes [27]. Phenotyping at 24–28 weeks in 554 women distinguished predominant beta-cell dysfunction, predominant insulin resistance and a mixed phenotype; the mixed group had higher BMI and more adverse outcomes in unadjusted analyses, but these differences did not survive adjustment for age and BMI [28]. Heterogeneity within GDM is therefore demonstrable, whereas phenotype-specific risk independent of adiposity is not, and neither finding can be transferred to eGDM without class A confirmation.

First-trimester triglyceride-glucose (TyG) indices point to a lipid-linked component of early insulin resistance but discriminate only modestly (class B). In a prospective Southeast Chinese cohort, each 1-unit increase in TyG was associated with a 3.63-fold higher risk of GDM (area under the curve 0.647) [35]. In 1624 women sampled before 14 weeks, each 1-unit rise carried a 2.10-fold increase in risk after adjustment for pre-pregnancy BMI and other covariates, and TyG alone gave an area under the curve of 0.641, rising to 0.672 when combined with maternal age and pre-pregnancy BMI [36]. Meta-analysis confirms the direction of association but shows that TyG alone is not accurate enough for diagnosis [57].

**Figure 1:**
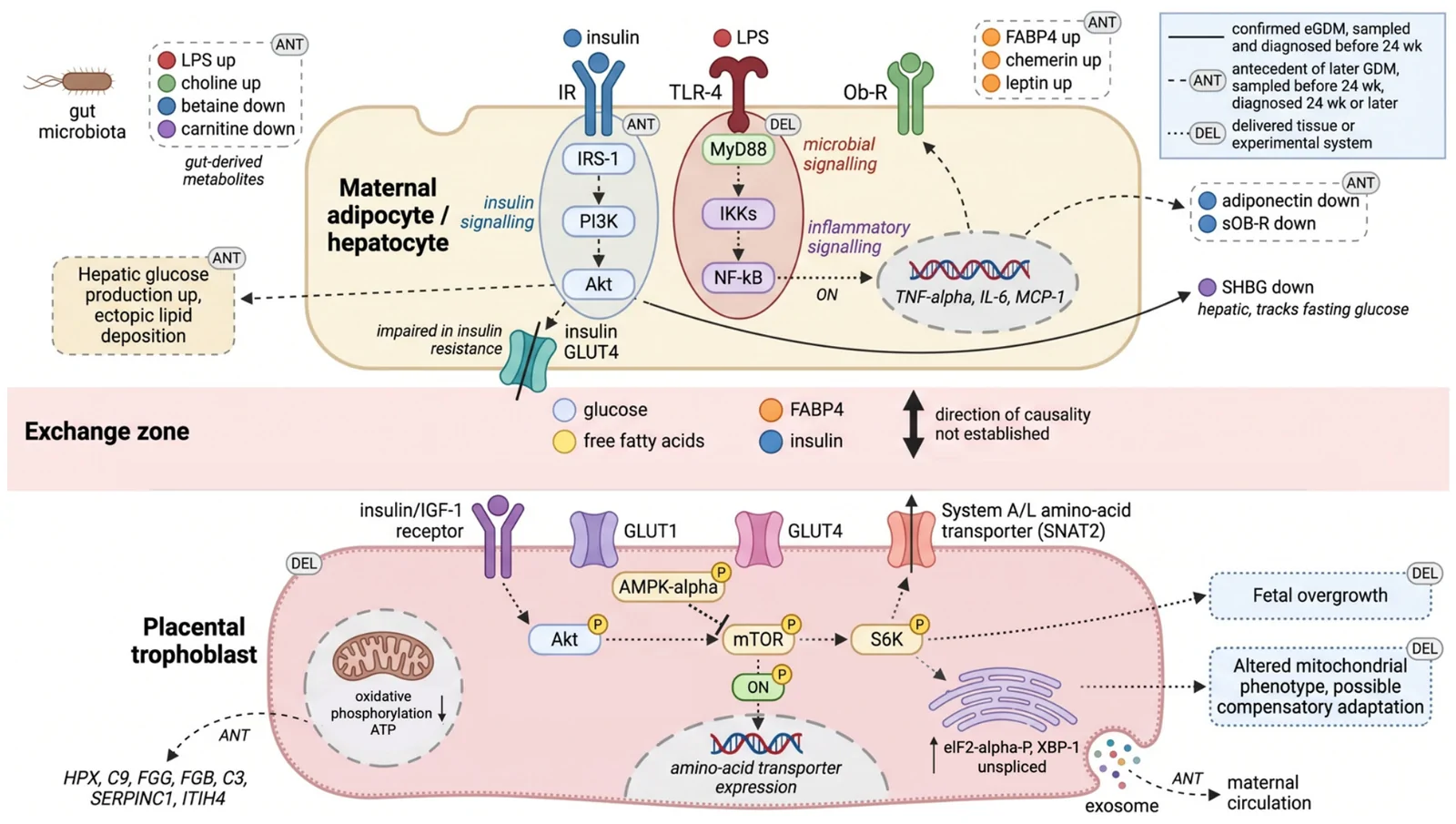
Molecules and cell types measured in and around the eGDM window. Schematic of the maternal hepatocyte and adipocyte compartment, the maternal-placental exchange zone and placental trophoblast. Line style encodes the class of evidence: solid for findings demonstrated in women diagnosed before 24 weeks, dashed for findings measured before 24 weeks in women diagnosed later, and dotted for delivered tissue or experimental systems. Abbreviations: AMPK-α, AMP-activated protein kinase α; Akt-P, phosphorylated Akt; C3, complement component 3; C9, complement component 9; eIF2-α-P, phosphorylated eukaryotic initiation factor 2α; FABP4, fatty acid-binding protein 4; FGB, fibrinogen β chain; FGG, fibrinogen γ chain; GLUT1/GLUT4, glucose transporter 1/4; HPX, hemopexin; IGF-1, insulin-like growth factor 1; IKK, IκB kinase; IL-6, interleukin-6; IR, insulin receptor; IRS-1, insulin receptor substrate 1; LPS, lipopolysaccharide; MCP-1, monocyte chemoattractant protein-1; MyD88, myeloid differentiation primary response 88; NF-κB, nuclear factor κB; Ob-R, leptin receptor; PI3K, phosphoinositide 3-kinase; S6K-P, phosphorylated ribosomal protein S6 kinase; SHBG, sex hormone-binding globulin; sOB-R, soluble leptin receptor; SNAT2, sodium-coupled neutral amino acid transporter 2; TLR-4, Toll-like receptor 4; TNF-α, tumour necrosis factor α. Created in BioRender. Baibussunova, A. (2026) https://BioRender.com/gbg0ohx (accesed on 26 September 2026)

Hepatic sex hormone-binding globulin (SHBG) production is suppressed by insulin and provides a liver-derived readout separable from adipokine and lipid measures. A nationwide Finnish case-control study measured SHBG, testosterone and the free androgen index at a mean of 10.7 weeks in 1045 women with GDM and 963 controls (mixed: GDM diagnosed at any gestation). After adjustment for gestational age at sampling, pre-pregnancy BMI and age, women with GDM had 3.7% (95% CI 0.1–7.3%) lower SHBG, 3.1% (0.1–6.2%) higher testosterone and 4.6% (1.9–7.3%) higher free androgen index; SHBG was associated specifically with fasting glucose, and testosterone and the free androgen index with post-prandial glucose (Figure 1). Women diagnosed before 20 weeks had a further 6.7% (0.7–12.7%) lower SHBG than other women with GDM [34], one of the few adjusted class A estimates available. These data suggest, but do not establish, a fasting-dominant pattern tracked by low SHBG and a post-load pattern tracked by androgen excess.

Women who later develop GDM therefore have higher fasting insulin, triglycerides and TyG-type indices and lower adiponectin in early pregnancy (class B) [23,29,30,31,36]. Glucose is one measured component of this profile, not demonstrably the initiating one, and an early glucose value is most informative when interpreted together with BMI, prior GDM, family history, polycystic ovary syndrome, lipid profile and whether the abnormality is fasting or post-load.

## 7. Adipose Signalling and Inflammation

Adipose-tissue findings come almost entirely from tissue collected at delivery (class D): omental and subcutaneous adipose tissue from women with GDM showed larger omental adipocytes, lower capillary density and reduced IGFBP5 expression [58], consistent with limited adipose expandability but unable to provide insight on when the limitation arose.

Circulating adipokines have been measured in early pregnancy with adjustment for adiposity, but all such studies are class B. Adiponectin was lower in 41 women who later developed GDM than in 70 controls (4.4 versus 8.1 µg/mL), and values below 6.4 µg/mL carried a 4.6-fold higher adjusted risk (95% CI 1.8–11.6) [29]. In 445 women, lower first-trimester adiponectin remained associated with GDM after adjustment for BMI and HbA1c (odds ratio 1.12 per 1 µg/mL decrease) [30]. In the NICHD Fetal Growth Studies, adjustment for age, gestational week, parity and family history of diabetes left higher FABP4 (odds ratio 4.87, highest versus lowest quartile), lower adiponectin (0.13) and lower soluble leptin receptor (0.24) at 10–14 weeks associated with later GDM, and most associations persisted, slightly attenuated, after further adjustment for pre-pregnancy BMI; adding FABP4, chemerin, soluble leptin receptor and high-molecular-weight adiponectin to age, race/ethnicity, family history of diabetes and random glucose, without BMI, raised the area under the curve from 0.71 to 0.77 [31]. These associations identify antecedents of later GDM; whether the same adipokines differ in eGDM diagnosed before 24 weeks has not, to our knowledge, been reported.

Inflammatory findings are directionally consistent but not specific. In healthy pregnancy, circulating TNF-alpha was the strongest independent correlate of insulin sensitivity after adjustment for fat mass [32]. Early-pregnancy leukocyte counts were higher in nulliparous women who later developed GDM (10.5 ± 2.2 versus 9.2 ± 2.2 × 10^9^/L; class B) [59], and at delivery 8-isoprostane release from placenta, adipose tissue and skeletal muscle were greater in GDM without differences in basal cytokine release (class D) [33]. None of these markers distinguishes GDM from other adiposity-related inflammation, so analyses require adjustment for pre-pregnancy BMI.

## 8. Maternal Diet and Gut-Microbial Metabolites

The evidence on diet depends on how dietary patterns are derived. A systematic review of 28 observational studies in 39,735 pregnant women found no definitive association between a posteriori-derived patterns and GDM risk [60], whereas a meta-analysis of ten studies reported lower odds of GDM with adherence to a Mediterranean-style diet before or during early pregnancy [61]. Neither approach has been evaluated in class A cohorts.

Gut microbial composition differs between women who do and do not develop GDM, with inconsistent direction for individual taxa. A review of 96 studies described dysbiosis-associated changes in short-chain fatty acid and bile-acid production, barrier integrity and lipopolysaccharide-linked inflammatory signalling, and some changes, including an altered Firmicutes/Bacteroidetes ratio and enrichment of *Bacteroides vulgatus* and *Ruminococcus gnavus*, are detectable in the first trimester [41]. Prospective sampling showed microbiota and inflammatory differences months before diagnosis (class B) [40].

Microbial metabolites give more directly quantifiable associations. In a nested case-control study of 321 GDM cases and 1284 age-matched controls (class B), plasma choline, betaine and carnitine were measured at 12–16 weeks and GDM was diagnosed at 24–28 weeks. After adjustment for early-pregnancy BMI and other covariates, each standard-deviation increase in choline carried a 16% higher risk of GDM (odds ratio 1.16, 95% CI 1.01–1.34), whereas betaine (0.81, 0.70–0.95) and carnitine (0.80, 0.69–0.94) carried lower risk; trimethylamine-N-oxide showed no association [39]. Methods across this literature are heterogeneous; no study relating diet or microbial metabolites to hyperglycaemia diagnosed before 24 weeks was identified, and because these associations overlap with adipokine and lipid measures, they should be collected alongside measures of insulin sensitivity and secretion.

## 9. Placental Nutrient Signalling and Trophoblast Stress: Indirect Mechanistic Evidence

Placental glucose flux depends on maternal glucose concentration, transporter abundance, uteroplacental blood flow and placental structure [62]. Placental glucose transport capacity and GLUT1 expression are altered in insulin-dependent diabetes [63], and GLUT4 is expressed in first-trimester syncytiotrophoblasts of normal pregnancy [42], so transport mechanisms are gestation- and cell-specific. Term placentas from diet-controlled and insulin-treated GDM show altered insulin-receptor-substrate and PI3K/AKT pathway proteins [44]. In primary trophoblasts, insulin and IGF-I increased system A amino-acid transport in an mTOR-dependent manner [64]; placental mTOR signalling, system A activity and SNAT2 expression correlated with birth weight across a wide maternal BMI range [65]; and placentas from GDM pregnancies with large-for-gestational-age infants showed higher Akt and mTOR phosphorylation and lower AMPK-alpha phosphorylation [66]. These associations are compatible with a contribution to foetal overgrowth, but their timing relative to maternal hyperglycaemia is unknown.

Trophoblast stress responses differ by lineage and milieu. GDM cytotrophoblasts showed lower oxidative phosphorylation and ATP production with lipid-droplet accumulation, changes not matched in syncytiotrophoblasts [45], and mitochondria isolated from the two lineages were affected, with different patterns and magnitude [48]. Term placentas from medication-treated GDM showed reduced miR-143 and mitochondrial respiratory-complex expression with increased GLUT1 and glycolytic enzymes, and trophoblast respiration was partly restored by miR-143 overexpression [46]; because treatment tracked severity, these data describe established GDM. A shift toward mitochondrial fusion has been interpreted as compensatory [67]. GDM syncytiotrophoblasts also showed endoplasmic-reticulum dilation and eIF2-alpha phosphorylation without XBP-1 splicing, and in trophoblast-like cells, the stress response to high-glucose culture was driven largely by medium acidification rather than glucose itself [47]. A 2026 systematic review of 56 high-throughput placental studies found no universal placental marker [68], and this search identified no lineage-separated placental data obtained before 20 weeks in eGDM.

The findings in this section derive from placentas collected at delivery, mostly after conventional-window GDM, and from trophoblast cultures and animal models, and none can establish whether a placental abnormality precedes, accompanies or results from maternal hyperglycaemia before 24 weeks.

## 10. Placenta-Derived Extracellular Vesicles

Extracellular vesicles allow the placenta to be sampled from maternal blood. Placental vesicles are detectable from 6 weeks and increase across the first trimester of normal pregnancy [69]. Placenta-derived exosome concentrations were higher in GDM by approximately 2.2-fold, 1.5-fold and 1.8-fold in the first, second and third trimesters (class B for first-trimester samples), and GDM exosomes induced greater endothelial cytokine release [70]; circulating exosomal proteomics in GDM identified 78 differentially abundant proteins in energy-production, inflammatory and metabolic pathways [71]. In functional experiments (class D), GDM placental exosomes reduced insulin-stimulated glucose uptake in skeletal-muscle cells [72], and small extracellular vesicles from GDM pregnancies impaired glucose homeostasis in mice [73], showing that vesicle cargo can alter maternal glucose handling but not that it precedes human eGDM.

The most relevant early-pregnancy dataset (class B) sampled total and placenta-derived plasma exosomes at 12–16 weeks in women who later developed GDM and matched controls, with repeat sampling at 24–28 weeks. Early placenta-derived exosomal proteins mapped to complement activation, fibrinolysis, platelet degranulation and coagulation, with hub proteins including HPX, C9, FGG, FGB, C3, SERPINC1 and ITIH4 [43]. Cohorts remain small, isolation methods differ and placental and non-placental vesicles are incompletely separated, so these candidates require standardised reporting and evidence of added value over maternal age, BMI and glucose.

## 11. Circulating Proteomic and Metabolomic Signatures

Proteomic studies performed before diagnosis (class B) converge on complement, coagulation, extracellular-matrix and lipid-related pathways while disagreeing on individual proteins. Serum profiling at 8–14 weeks in 60 women with a BMI of at least 27 kg/m^2^ identified 25 differing proteins; afamin, serum amyloid P-component and vitronectin were confirmed in a second sample set of 210 women from the same population, and vitronectin moved the area under the curve of a maternal risk-factor model from 0.798 to 0.806 [49]. Comparisons of 30 cases and 30 controls at 12–16 weeks yielded a four-protein panel with an area under the curve of 0.985 in the same sample [52]; CRP and IGFBP2 identified at 16–18 weeks reached 0.953 and 1.00 in 15 further cases and 15 controls [53]; and thrombospondin-4 and CNDP1, discovered in five cases and five controls, reached 0.94 and 0.98 in 25 cases and 25 controls [54].

Metabolomic studies identify early disturbances in lipid and central-carbon pathways. One study profiled more than 150 lipid species and central-carbon intermediates in first-trimester plasma from 20 women with early-onset GDM, identified by first-trimester Carpenter–Coustan screening of high-risk women (class A), 20 with late-onset GDM (class B) and 20 controls; both GDM groups showed abnormalities, with several triglycerides, including TG(46:2), markedly increased in late-onset GDM [74]. In 107 women with GDM and 107 matched controls, 31 metabolites were altered in the first trimester after false-discovery-rate correction [75]. Other cohorts describe broad perturbations rather than a reproducible single marker, in HAPO (class C) [76], in women with obesity before diagnosis [37] and in a nested early-pregnancy analysis [77], and a two-acylcarnitine model with an area under the curve of 0.934 included only 13 GDM cases [78]; urinary candidates were reviewed separately [79].

Two linked urinary studies address gestational-age specificity in the same women. Early-pregnancy urine (mean 12 weeks, range 6–19) from 46 GDM cases and 46 controls matched for age, pre-pregnancy BMI and gestational age yielded a seven-metabolite model with 96.7% accuracy (class B) [50]. In urine collected at or after 24 weeks (class C), a four-metabolite tree identified GDM with 89.1% accuracy and a receiver-operating-characteristic area of 0.923, whereas the early-pregnancy model applied to late urine gave 85.9% [51]. Only partial overlap between windows, with saccharopine associated with GDM in both, indicates that panels are gestational-age-specific and must be re-derived for early gestation.

The discrimination reported in these studies is exploratory. Most panels were derived in case-control samples of fewer than 100 women, report performance in their development samples, did not adjust for or stratify by BMI, or handled it only by matching, and have not been tested in an independent cohort. Case-control sampling of clearly defined cases and controls also tends to overestimate discrimination relative to screening populations, in which lower prevalence would further reduce positive predictive values. Areas under the curve approaching 1.0 in samples of this size are therefore a property of design as much as of biology, and no clinically validated early biomarker of eGDM currently exists.

## 12. Lipidomic Signature of Early-Diagnosed GDM

The TOBOGM lipidomic analysis of plasma from 180 women at risk of GDM, 89 with eGDM diagnosed before 20 weeks and 91 controls, quantified 543 lipid species across 18 classes. eGDM was associated with higher diacylglycerols, fatty acids, phosphatidylethanolamines and lysophosphatidylethanolamines and lower lysophosphatidylcholines, ether-linked phosphatidylcholines and hexosylceramides, and fatty-acid and diacylglycerol species remained associated with eGDM and glycaemic indices after adjustment for age, BMI, gravidity, gestational week, ethnicity and family history of diabetes [38]. Its strengths, namely sampling and diagnosis in the same pre-20-week window, diagnosis by a prespecified OGTT standard within a randomised trial, and adjustment that includes BMI, are those most other studies in this field lack.

Three cautions apply. The findings come from a single trial cohort selected for GDM risk factors; they have not been replicated in an independent eGDM population or on another platform; and because lipids and glycaemia were assessed in the same window, the analysis cannot show whether the lipid changes preceded hyperglycaemia.

## 13. Which Early Markers Remain Associated After Adjustment for Adiposity

There remain the questions of whether an association survives adjustment for adiposity and how well a marker classifies women with GDM. HOMA-IR, adiponectin, soluble leptin receptor, FABP4, SHBG, the free androgen index, TyG, choline, betaine and carnitine remain associated with GDM after adjustment for, or stratification by, pre-pregnancy or early-pregnancy BMI, but these estimates are class B or mixed and describe adjusted antecedents of later GDM [23,30,31,34,36,39]. The TOBOGM lipid species and the SHBG estimate in women diagnosed before 20 weeks are the only BMI-adjusted class A estimates identified [34,38]. In the urinary metabolomic studies, adiposity was handled by matching, which reduces but does not eliminate residual confounding [50,51]. For classification, vitronectin adds a small increment to maternal risk factors [49], TyG alone discriminates modestly (area under the curve 0.641, or 0.672 with age and BMI) [36], and the discovery proteomic panels report high discrimination in small samples without BMI adjustment [52,53,54].

Evidence classes as defined in Section 3 and Table 1. Not available indicates that the information could not be obtained from the source publication; not reported indicates that the authors did not report it. Abbreviations: AUC, area under the curve; CI, confidence interval; FAI, free androgen index; GA, gestational age; OR, odds ratio; ROC, receiver operating characteristic; SD, standard deviation; sOB-R, soluble leptin receptor; other abbreviations as in Table 1.

## 14. Clinical Implications

The molecular and metabolic evidence reviewed here currently has no direct application to the diagnosis or management of eGDM. Clinical decisions in early pregnancy rest on glucose testing: TOBOGM supports the diagnosis and treatment of GDM identified before 20 weeks by the WHO 2013 criteria in women with risk factors [9], and ADA guidance distinguishes overt diabetes, early abnormal glucose metabolism and conventional screening at 24–28 weeks [16].

To change practice, a biomarker would need reproducible associations across cohorts and platforms, discrimination additional to maternal age, BMI, prior GDM, family history and early glucose, independence from adiposity, external validation in a population with realistic prevalence and, ultimately, evidence that its use improves outcomes. None of the candidates reviewed here meets these criteria (Table 2). Class B markers discriminate modestly: an adipokine panel raised the area under the curve from 0.71 to 0.77 when added to age, race/ethnicity, family history and random glucose [31], and TyG alone reached 0.641, or 0.672 with age and BMI [36]; panels with very high reported discrimination have not been externally validated [50,51,52,53,54,78]; and the only class A molecular signature has not been replicated [38]. The evidence therefore remains exploratory.

Three practical points follow. Because about one-third or more of early diagnoses regress by 24–28 weeks [13,18], a marker that distinguished persistent from transient early hyperglycaemia would be more useful than one that merely predicted GDM. Fasting hyperglycaemia at a normal BMI, particularly with a parental history of mild diabetes, should prompt consideration of GCK-MODY [55]. In addition, readily available clinical information, namely BMI, early glucose severity and whether hyperglycaemia is fasting or post-load, should be reported in any study proposing a new marker, because candidate molecules have so far added only modestly to the research.

## 15. Forward-Looking Outlook

The evidence identifies candidate contributors to eGDM at three levels: maternal metabolic, dietary and microbial exposures and circulating signatures measured before 24 weeks, which are largely class B; a small body of class A data from women diagnosed before 20 weeks; and placental and trophoblast findings from delivered tissue or experimental systems (class D). Their order is not established, eGDM may comprise several parallel phenotypes converging on hyperglycaemia, and any integration of these findings into a single insulin-resistant pathway should be treated as a hypothesis.

The design most likely to resolve these questions is a longitudinal cohort in which women are phenotyped before conception or at first antenatal contact and repeatedly during pregnancy, with repeated glucose assessment including an early OGTT, with fasting and post-load values reported separately, and retesting at 24–28 weeks; dynamic measurement of insulin sensitivity and secretion, by clamp, intravenous glucose tolerance testing or validated OGTT-derived indices, so that a disposition index can be calculated at each time point; direct measures of adiposity, with prespecified adjustment; molecular profiling of plasma and extracellular vesicles at matched time points; and testing for monogenic hyperglycaemia where indicated. Such a cohort would distinguish persistent eGDM, transient early hyperglycaemia, conventional GDM preceded by early metabolic abnormalities and normal glucose tolerance and would show which molecular changes precede hyperglycaemia and which follow it (Table 3).

The limiting factor in this field is not a shortage of candidate molecules but a shortage of studies designed around the pre-24-week window. Phenotype-stratified trials using the TOBOGM enrolment window could test whether insulin-resistant, secretion-limited and other subgroups respond differently to treatment [9]. Until such data are available, statements about the molecular pathogenesis of eGDM should remain statements of association, and early-pregnancy biomarkers should be regarded as research tools rather than clinical tests.

## Figures and Tables

**Table 3:** Study characteristics required to establish sequence rather than association in eGDM.

Characteristic	What It Means in Practice	Why Present Evidence Is Insufficient
A single operational definition	Hyperglycaemia diagnosed before 24 weeks using established criteria, without overt diabetes, with fasting and post-load values reported separately	Cohorts currently mix first-trimester, <20-week and <24-week diagnoses and different glucose thresholds, so estimates are not comparable [13,16]
Serial phenotyping before and during pregnancy	Repeated measurements from pre-conception or first antenatal contact to 24–28 weeks in the same women	Most studies sample once, so changes cannot be ordered relative to the onset of hyperglycaemia
Retesting at 24–28 weeks	Repeat testing in the same women so that persistent, transient and late-emerging hyperglycaemia can be separated	About one-third of women diagnosed before 20 weeks in TOBOGM, and up to one-half in other cohorts, are normoglycaemic on retesting, and cohorts rarely report the proportion [13,18]
Dynamic assessment of secretion against sensitivity	Clamp, intravenous glucose tolerance test or validated OGTT-derived indices combined as a disposition index before 20 weeks	Class B studies used fasting surrogates (HOMA-IR, HOMA-beta, QUICKI) [23]; the class A DALI analysis used HOMA-IR with the OGTT-derived Stumvoll first-phase index, which estimated post-load secretion but was not referenced to concurrent sensitivity [25]; one small early-GDM group was assessed with an OGTT-derived disposition-type index [24]; clamp and intravenous glucose tolerance testing were applied at 12–14 weeks in women with and without later GDM [22], but this research did not identify their use specifically in women with eGDM diagnosed before 20 weeks
Exclusion of monogenic hyperglycaemia	Consideration of GCK-MODY where fasting hyperglycaemia occurs at normal pre-pregnancy BMI with a parental history	An estimated 1.5% of women labelled as having GDM meet criteria for suspected GCK-MODY, which does not share acquired metabolic mechanisms [55]
BMI-adjusted reporting	Effect estimates adjusted for, or matched on, pre-pregnancy BMI for every candidate biomarker, with covariates listed	Several high-discrimination proteomic panels report no adjustment [52,53,54], and covariates are incompletely reported for others (Table 2)
Independent replication and validation	Replication of class A findings and external validation of prediction models in independent cohorts	The TOBOGM lipidome was not replicated [38], and proteomic and metabolomic models were reported only in development samples [49,50,51,52,53,54]
Cell-specific placental sampling	Cytotrophoblast and syncytiotrophoblast analysed separately	Nearly all placental data are from delivered tissue [45,47,48]
Diet and microbiome measured alongside metabolic indices	Dietary quality scores, microbial composition and microbial metabolites collected with insulin sensitivity and secretion measures in the same women	Diet and microbiota associations overlap with adipokine and lipid measures, and their independence from them was not tested in eGDM [39,41,60,61]

Abbreviations: OGTT, oral glucose tolerance test; GCK-MODY, glucokinase maturity-onset diabetes of the young.

## Data Availability

No new data were created or analysed in this study. Data sharing is not applicable to this article.
